# Germ-line *DICER1* mutations do not make a major contribution to the etiology of familial testicular germ cell tumours

**DOI:** 10.1186/1756-0500-6-127

**Published:** 2013-04-01

**Authors:** Nelly Sabbaghian, Amin Bahubeshi, Andrew Y Shuen, Peter A Kanetsky, Marc D Tischkowitz, Katherine L Nathanson, William D Foulkes

**Affiliations:** 1 Program in Cancer Genetics, Department of Oncology and Human Genetics, McGill University, Montreal, QC, Canada; 2 Lady Davis Institute and Segal Cancer Centre, Jewish General Hospital, McGill University, Montreal, QC, Canada; 3 Department of Human Genetics, McGill University, Montreal, QC, Canada; 4 Department of Biostatistics and Epidemiology, Perelman School of Medicine at the University of Pennsylvania, Philadelphia, PA, USA; 5 Division of Translational Medicine and Human Genetics, Department of Medicine, Perelman School of Medicine at the University of Pennsylvania, Philadelphia, PA, USA; 6 Abramson Cancer Center, Perelman School of Medicine at the University of Pennsylvania, Philadelphia, PA, USA; 7 Research Institute, McGill University Health Centre, Montreal, QC, Canada; 8 Current Addresses: Faculty of Medicine, University of Toronto, Toronto, ON, Canada; 9 Department of Medical Genetics, University of Cambridge, Addenbrooke’s Hospital, Level 6, Addenbrooke’s Treatment Centre Box 134, Cambridge, CB2 0QQ, UK

**Keywords:** microRNA, Germ cell tumors, Dicer, Mutation analysis, High-resolution melt analysis

## Abstract

**Background:**

The RNase III enzyme DICER1 plays a central role in maturation of microRNAs. Identification of neoplasia-associated germ-line and somatic mutations in *DICER1* indicates that mis-expression of miRNAs in cancer may result from defects in their processing. As part of a recent study of DICER1 RNase III domains in 96 testicular germ cell tumors, a single RNase IIIb domain mutation was identified in a seminoma. To further explore the importance of *DICER1* mutations in the etiology of testicular germ cell tumors (TGCT), we studied germ-line DNA samples from 43 probands diagnosed with familial TGCT.

**Findings:**

We carried out High Resolution Melting Curve Analysis of *DICER1* exons 2–12, 14–19, 21 and 24–27. All questionable melt curves were subjected to confirmatory Sanger sequencing.

Sanger sequencing was used for exons 13, 20, 22 and 23. Intron-exon boundaries were included in all analyses. We identified 12 previously reported single nucleotide polymorphisms and two novel single nucleotide variants. No likely deleterious variants were identified; notably no mutations that were predicted to truncate the protein were identified.

**Conclusions:**

Taken together with previous studies, the findings reported here suggest a very limited role for either germ-line or somatic *DICER1* mutations in the etiology of TGCT.

## Introduction

Animals and plants express hundreds of miRNAs, which are predicted to target and regulate at least 60% of protein-coding mRNAs and are integral to almost all known biological processes. DICER1 is highly conserved throughout evolution, and contains several functionally important domains. We and others have identified both germ-line and somatic mutations in *DICER1* that are associated with a range of mainly childhood-onset cancers and dysontogenic or hyperplastic conditions, notably “blastoma”-type tumors such as pleuropulmonary blastoma (PPB), ovarian Sertoli- Leydig cell tumor (SLCT), embryonal rhabdomyosarcoma and Wilms tumor, as well as benign tumors such as cystic nephroma [1-10]. Despite a detailed study of hundreds of cancer cell lines [4], the full extent and limit of the involvement of both germ-line and somatic *DICER1* mutations in rarer types of human cancer is currently unknown.

A large study of all exons of *DICER1*, conducted using DNA from 4 microsatellite-stable testicular germ cell tumor (TGCT) cell lines and germ-line DNA from 185 persons with a germ cell tumor (of whom 71 had a seminoma and 128 of whom had a family history of TGCT) revealed one germ-line mutation, c.4740G > T, p.Q1580H, in a man with a past personal history of seminoma [4]. The mutation is of unknown significance, but according to Polyphen2 [11], this mutation is predicted to be probably damaging with a score of 0.996 (sensitivity: 0.55; specificity: 0.98), and in agreement with this, SiftBLink [12] indicates that substitution at position 1580 from Q to H is predicted to affect protein function with a score of 0.00 (less than 0.05 is usually regarded as evidence for a deleterious effect on protein function).

Recurrent “hotspot” somatic mutations exist in the RNase IIIb domain of DICER1 [10]. These hotspot mutations were mainly identified in SLCT, but of 26 TGCT analysed for the hotspots, a single non-seminomatous TGCT was found to possess c.5125G > A, p.D1709N. It could not be determined if the mutation was germ-line or somatic in nature [10], but this mutation is functionally deleterious [13] and therefore could be etiologically related to the occurrence of the TGCT. Another study did not identify a *DICER1* mutation in a man with a seminoma, who was the relative of a patient with a PPB [5].

Recently, de Boer et al. reported finding only one presumed somatic RNase IIIb domain mutation (c.5174G > A; p.R1725Q) among 96 TGCT for mutations in this domain [14]. Bioinformatic analysis of this variant gives varying results; whereas Polyphen2 [11] suggests that this mutation is predicted to be probably damaging with a score of 1.000 (sensitivity: 0.00; specificity: 1.00), SiftBLink [12] reports that substitution at position 1725 from R to Q is predicted to be tolerated with a score of 0.18.

In view of these previous studies, we wished to establish if germ-line *DICER1* mutations play a role in the etiology of TGCT, with the clinical aim of better counselling *DICER1* mutation carriers as to their cancer risks. We report here our analysis of germ-line DNA from 43 probands with a personal and family history of TGCT.

## Methods

### Subjects

Men with TGCT were recruited through an on-going case–control study at the Perelman School of Medicine at the University of Pennsylvania, which has been previously described [15,16]. All patients completed a questionnaire, which includes self-reported information about family history of TGCT. For the current study, men with TGCT who reported a family history of at least one relative also with TGCT were selected (Table 1). All studies were carried out in accordance with the Institutional Review Board (IRB) of the University of Pennsylvania with written consent (IRB study number: 703123).

**Table 1:** Type of testicular germ cell tumor in 39 probands^#^ plus degree of relatedness to familial case(s) of TGCT

	**Degree of relatedness to affected relative**				
**Type of testicular germ cell tumor in the proband**	**1**^**st**^	**2**^**nd**^	**3**^**rd**^	**>3**^**rd**^	**Total**
Seminoma, NOS	5	3	4	0	12
Embryonal carcinoma, NOS	1	3	1	1	6
Teratoma, benign	0	0	1	0	1
Teratocarcinoma	1	0	2	2	5
Choriocarcinoma combined with other germ cell elements	0	0	2	0	2
Yolk sac tumor	0	0	1	0	1
Germ cell tumor, nonseminomatous	0	0	1	0	1
Mixed germ cell tumor (mixed teratoma and seminoma)	1	1	0	0	2
Mixed germ cell tumor (mixed embryonal and seminoma)	3	1	2	0	6
Mixed germ cell tumor (mixed yolk sac and seminoma)	0	0	0	1	1
NSGCT (mixed yolk sac and teratoma, benign)	0	0	1	0	1

*First degree- parent, sib, child; second degree - aunt, uncle, grandparent, grandchild; third degree – cousin; more than third – second cousin. If more than one relative affected, only the closest degree of relatedness is included.

NSGCT – Non-seminoma germ cell tumor; NOS – not otherwise specified.

^**#**^Family history could not be confirmed for two cases (seminoma and yolk sac tumor) and pathology could not be fully confirmed on a further two cases. These four cases were excluded from the table.

#### Experimental details

We conducted High Resolution Melting Curve (HRM) analysis of *DICER1* [GENBANK NM_177438.2**]** exons 2–12, 14–19, 21, the 3’ half of 23 and 24–27 using lymphocyte DNA from one proband from each family as described previously [3]. Briefly, we screened 22 of the 26 coding exons of *DICER1* by (HRM) using the LightScanner instrument (Idaho Technologies Inc., Utah, USA). The PCR reactions were done in 96 well plates from Bio Rad (Ontario, Canada) using the mastermix and the LCGreen Plus from Transition Technologies (Ontario, Canada). The plates were then transferred to the LightScanner instrument and the melted curves were analyzed by the software provided by Idaho Technologies. This technique was used as a presequencing selection for amplicons harboring variants. The PCR primers used are shown in Table 2. All questionable melt curves were subjected to confirmatory Sanger sequencing, which due to the complexity of the HRM results, was used as the sole method of *DICER1* analysis for exons 13, 20, 22 and the 5’ half of exon 23. Intron-exon boundaries were included in all analyses.

**Table 2:** DICER1 oligonucleotide primers used in this study

**Exon**	**Forward**	**Reverse**	**Size of fragment**	**Annealing temp. Sanger sequencing**	**Annealing temp. HRM**
2	GCAATGAAAGAAACACTGGATG	TCAAATCCAATTACCCAGCAG	358	[1]	64
3	TTTTGTAAATTTATTGGAGGACG	TCTGCCAGAAGAGATTAAATGAG	429	[1]	64
4	TTTTGGAGGATAACCTTGGAAC	AAATCAGACAACCAAGGCTACAG	390	[1]	66
5	TTGTCGTCAAGACATGCTTTC	TTTAATATTCATTCATTCATACACTGC	518	[1]	66
6	TAGTGGCATTTCCACCAAAC	GAATTCTTACTCTTGCCCATTCC	437	[1]	69
7	TTCTCACTACTGCAGTATTGATACCTT	GAGCCGCATTAAGCATATTTTC	303	[1] (7 F modified)	69
8	AAATCCCAGTTAAACCCCAC	TCACATCACAACACAGGACG	554	[1]	68
9	TAAATCACCGTCGCCAAATC	AAATCACTCTACAGCTACCTCATGG	591	[1]	69
10	CATGTGTGTCAGAAATGACAGTTG	TTCCTATGGATACAAAGAATAACAAAG	431	[1]	68
11	AGCAGGTTACTTTGGAGTACTGAAG	AACTTTTATTGCTGCACGATACTG	498	[1]	69
12	TCACATTTCAAGTGCTCACC	TGAACATGTAGATGACTACAAAAGC	596	[1]	69
13	TTTTACTAGGCAGGACTTTTAAAGATG	AAGTGTTCATGGTGCATGATTC	585	[1]	NA
14	TTTGCAGTCCAGCTCATATTG	AAGCTGTGAATCGGAGAAAG	498	[1]	69
15	TAAGAAGTGTCATGCCTCGG	TCTAGTGGAGAAATAGAAGAGGCAC	468	[1]	68
16	GAAAGCATCATTTCTGTTCTGAAG	AAGAGAAAAACGACTCTTTAGC	443	16R is new	65
17	TTCAGCATACTGTGTTCTACCTCTT	TTTTAGTAGAGACGAGGTTTCACC	484	17 F is new	69
18	TGTAAAGGTGCCATTTAGCTTC	TTTGTGTGCAAAGCATCTCC	589	[1]	69
19	ATTGCACTTGAGGGATTCTTACC	TTTGTGATATATTAATGGGCCAAG	496	[1]	67
20	TTGGCCCATTAATATATCACA	TCTCACTCCAACTGTTATGGCTTA	594	[1]	NA
21-1	AATTGCTGTTGCTCTCAGCC	GAGTACATTCATCGCTGGGC	508	[1]	68
21-2	ACAAGCAGGAAATACCCGTG	ACTGCAAACCACTTTCAGGC	501	[1]	68
22	AAAGCATAGAATATGTGGGAATT	AGAAATTTGCCTCCATCAAA	584	[1]	NA
23-1	AACCCTTGCTTTTATTGAGTTTC	CAGGGCTTCCACACAGTCC	574	[1]	NA
23-2	AAACTGTGGTGTTGACACGG	TACAAGGCCAACACGATGAG	571	[1]	68
24	TGTGGGGATAGTGTAAATGCTTC	TGCCGTCAGAACTCTGAAAC	403	[1]	68
25-26	TGGACTGCCTGTAAAAGTGG	TGAACTTTTCCCCTTTGATG	450	[1]	66
27	CCTGTCTGTCGGGGGTATG	TCTGCCTTCAATTCATTCCA	448	[1]	69

Key: HRM- High Resolution Melt. Oligonucleotide primers 16R and 17 F are new; they were designed specifically for High Resolution Melt analysis.

NA: Not Applicable – the fragments amplified were not included in the HRM assay, they were sequenced instead.

[1] refers to the source of the oligonucleotide primer sequences. bp - base pairs.

### Findings

Among the 43 probands, we identified 14 different single nucleotide variants or polymorphisms, 12 of which have been previously reported, but no likely deleterious variants; notably no mutations that were predicted to truncate the protein were identified (Table 3).

**Table 3:** ***DICER1 *variants observed in 43 TGCT probands studied 14 samples had no SNPs**

**Variant**	**Predicted function**		**Number of cases**
c.1377-25 T > A	Likely non-pathogenic	Previously reported	1****
c.1509 + 32A > G, rs144973109	Likely non-pathogenic	Previously reported	2
c.1907 + 43C > T, rs11624081	Likely non-pathogenic	Previously reported	6*
c.1907 + 105C > T, rs2275182	Likely non-pathogenic	Previously reported	2*
c.1935G > A p.P645P, rs61751177	Likely non-pathogenic	Previously reported	2^**§**^
c.2041-91A > G, rs2297730	Likely non-pathogenic	Previously reported	6, 1***, 1^**§§§**^
c.2116 + 59insA	Likely non-pathogenic	Novel	1***
c.2116 + 65A > T, rs187825570	Likely non-pathogenic	Previously reported	1**
c.2804 + 62C > T, rs117996122	Likely non-pathogenic	Previously reported	1^**§§**^
c.2805-129G > A	Likely non-pathogenic	Novel	1
c.2997 T > G p. L999L, rs12018992	Likely non-pathogenic	Previously reported	1***
c.3093 + 178 T > C, rs17091820	Likely non-pathogenic	Previously reported	1***
c.5145C > T p. L1715L, rs139500905	Likely non-pathogenic	Previously reported	1
c.*88 T > A, rs13078	Likely non-pathogenic	Previously reported	15

* one case has s11624081 in addition to this variant, and another has both rs61751177 and s11624081 (i.e. this person carries 3 variants).

** has both rs2297730 and rs13078 in addition to this variant.

*** has rs2297730, rs17091820, rs12018992 and c.2116 + 59insA (i.e. this person carries 4 variants).

**** has rs144973109 and rs2297730 in addition to this variant.

^**§**^ one case has both rs2275182 and rs11624081 in addition to this variant (i.e. this person carries 3 variants), and one case has rs13078 in addition to this variant.

^**§§**^ has rs13078 in addition to this variant.

^**§§§**^ has rs13078 in addition to this variant.

TGCT account for 1 percent of all malignancies in males, but are the most common cancer among young men aged 15–35 years. Most germ cell tumors can be classified as seminomas or non-seminomas, while a small proportion are of mixed histology. Established risk factors include cryptorochidism, previous diagnosis of TGCT, subfertility and family history of TGCT (reviewed in [17,18]).

Multiple epidemiological studies point toward a strong genetic basis for TGCT susceptibility. A large Swedish study estimates the genetic contribution of TGCT susceptibility to be about 25%, the third highest among cancers. Although familial aggregation of TGCT is rare with only 1.4% of families having two or more first degree relatives with the disease, multiple studies in different populations have shown that sons of an affected father are at 4–6 fold increased risk of developing TGCT while brothers of an affected male are at 8–10 fold increased risk, a familial relative risk that is much higher than most other cancers. Ethnic variability is also observed with an incidence five times higher in Caucasian males than in African-Americans (as reviewed by Rapley and Nathanson) [19].

A genome-wide linkage search for susceptibility loci initially identified a region on Xp27 as a possible candidate, however this finding was not replicated in an independent data set, the results of which suggested that no single highly penetrant allele is responsible for a substantial proportion of familial TGCT [20]. Candidate-gene analysis of a “gr/gr” deletion on the Y chromosome known to cause infertility, was found to be associated with a 2–3 fold risk of developing TGCT [21], however the deletion was present in only 2% of TGCT patients unselected for family history, explaining just 0.5% of the excess familial risk [22]. Other candidate-gene analyses have suggested associations with genes involved in immune and hormone regulation, however these findings have not been confirmed. Stronger evidence has come from recent genome-wide association studies that have identified six susceptibility loci implicating *KITLG, SPRY4, BAK1, TERT, ATF7IP and DMRT1* in disease pathogenesis. Nevertheless, these six loci together with the “gr/gr” deletion account for less than 15% of the excess familial risk, suggesting that many more risk alleles remain unaccounted for (reviewed in [19,22]). A recent study suggested a possible role for *de novo* germline copy number variants (CNVs); such variants were seen in 7% of 43 TGCT trios, greater than the expected background rate of CNVs [23]. With these results in mind, whole exome/genome sequencing studies, focusing on large series of familial TGCTs is likely to be the next step in efforts to understand the genetic basis of TGCT.

The findings reported herein, when combined with the previously reported studies discussed above, suggest that neither germ-line nor somatic *DICER1* mutations are commonly associated with TGCT. These results strongly suggest that TGCTs do not fall within the spectrum of diseases associated with germ-line *DICER1* mutations and thus clinical screening for such cancers is not warranted in *DICER1* mutation carriers.

## Abbreviations

CNVs: Copy number variants; PPB: Pleuropulmonary blastoma; SLCT: Sertoli-Leydig cell tumor; TGCT: Testicular germ cell tumor.

## Competing interests

The authors declare that they have no competing interests.

## Authors’ contributions

AB and NS did the HRM analysis and sequencing, AYS helped with interpretation of the results and writing of the paper, PAK and KLN ascertained the patients and maintained the database, MDT and WDF oversaw the project. WDF wrote the paper, which was edited by all authors, who commented on and approved the final version. All authors read and approved the final manuscript.
